# Opinion: Is Pain an Overlooked Patient Outcome? Elevating Post-Operative Pain Above a Footnote

**DOI:** 10.3389/fopht.2022.871976

**Published:** 2022-03-16

**Authors:** Maria Moustaqim-Barrette, Eric A. Moulton

**Affiliations:** ^1^ Department of Pediatrics, Boston Children’s Hospital, Harvard Medical School, Boston, MA, United States; ^2^ Brain and Eye Pain Imaging Lab, Pain and Affective Neuroscience Center, Department of Anesthesiology, Critical Care and Pain Medicine, Boston Children’s Hospital, Harvard Medical School, Boston, MA, United States; ^3^ Department of Ophthalmology, Boston Children’s Hospital, Harvard Medical School, Boston, MA, United States

**Keywords:** cornea, ocular surface, recovery, CXL, wound healing

## Introduction

Pain, especially if predictable and relatively short-lived, is often viewed as a temporary yet necessary side effect. The focus of most ophthalmological studies is on preserving and/or restoring vision, as it should be. However, over the past 15 years, other medical specialties such as general surgery have increasingly attended to the possibility of the development neuropathic post-surgical pain. Acute post-operative pain occurs in 10-50% of patients following common surgeries, and 2-10% of these go on to develop chronic pain (1). This increasing awareness of chronic pain risk following surgery has started to spread to ophthalmology as well, but the tracking and reporting of pain in clinical trials still has some ways to go. A recent comprehensive review of pain mechanisms in corneal collagen cross-linking (CXL) from our group calls attention to these challenges and highlights various areas for improvement in the assessment of ocular pain following this increasingly available treatment for keratoconus.

## Post-Operative Pain in Corneal Collagen Cross-Linking

Acute postoperative pain is a well-known complication of CXL. In a randomized controlled trial on CXL for progressive keratoconus, Hersh et al. (2) found that 17% of patients in the treatment group experienced acute eye pain, while other authors report acute pain in as high as 43% of cases (3). Interestingly, much less is known about the chronification of acute ocular pain following CXL and there is a notable absence of reported cases in the literature to date.

Van der Valk Bouman et al.’s review of pain mechanisms in CXL casts a spotlight on post-operative pain, and points to a lack of systematic evaluation of pain outcomes (4). Of the twenty-one studies on post-CXL pain that were reviewed, all were found to report acute pain occurring within 24 hours of the procedure. Persistent post-operative was investigated in three studies, none of which reported persistent post-procedural pain at time points ranging from two weeks to one year post-CXL. The authors examined what metrics were used for pain measurement within these studies and found significant variability in the scales used for the assessment of pain. Unidimensional scales such as the Visual Analog Scale (VAS), Numerical Rating Scale (NRS), and Wong-Baker FACES Pain Rating Scale (FACES) were used most frequently, and in some cases the tools used for pain measurement were not reported.

In addition to an analysis of pain measurements across studies, the paper identified several holes in our understanding of the chronification of pain following CXL and offered a number of possible explanations for these gaps in knowledge. Assuming chronic pain is an underdiagnosed consequence of CXL, van der Valk Bouman et al. point to a paucity of longitudinal studies measuring delayed onset of pain following CXL (4). Other possibilities explored by the authors included an underreporting of cases either due to the infrequency of this complication or to barriers in communication given that the procedure is frequently offered to pediatric and developmentally delayed patients. Conversely, should persistent post-procedural pain never develop following CXL, possible explanations for the mechanisms underlying the absence of chronification include potentially protective factors inherent to the target demographic, such as young age, and the surgical approach of the procedure itself whereby an incisionless technique spares deep nociceptive afferents

## Discussion

Growing evidence suggests that chronic neuropathic ocular pain is a largely underrecognized and frequently misdiagnosed complication of eye surgeries and procedures (5–7). The lack of chronic pain following CXL is intriguing, and provokes questions about the mechanism underlying this absence. On the other hand, if chronic pain is indeed a complication of this increasingly available treatment, how can we further characterize this problem in order to better serve patients?

As is evident from van der Valk Bouman et al.’s review, some of the challenges to better understanding post-CXL pain can be attributed to the variability in pain assessment and to the use of unidimensional pain scales within the literature. The use of unidimensional scales focuses solely on intensity while overlooking qualitative features of pain and its impact on quality of life. This confines pain to a nonspecific complaint, with frequently nebulous diagnostic utility rather than considering pain as an evolving and complex post-surgical event. Consistent use of a multidimensional pain assessment survey such as the Ocular Pain Assessment Survey (OPAS), Neuropathic Pain Survey Instrument-Eye (NPSI-Eye), or the McGill Pain Questionnaire (MPI) could facilitate both the identification and comparison of acute and persistent pain symptoms in a more comprehensive manner. For more expeditious yet still clinically meaningful measurement of pain, adoption of the validated VAS, commonly used in pain clinics and in research, would bolster cross-study comparisons and increase statistical fidelity. A standardized, systematic approach to studying pain following common corneal procedures would also be integral in developing best practices for the management of acute pain post-CXL which can be severe and difficult to control (3).

A more holistic approach to assessing pain could also facilitate the identification of features that can affect the transition from acute to chronic pain, such as neuropsychiatric conditions (8, 9). In their study on corneal pain following LASIK surgery, Moshirfar et al. (9) found that half of patients with persistent symptoms had neuropsychiatric conditions. While persistent pain symptoms remain to be identified post-CXL, further investigation into the role of these comorbid conditions following other common corneal procedures such as LASIK and PRK would enable early identification and timely follow-up of at risk patients in the perioperative period. More broadly, this could encourage an interdisciplinary approach to high risk patients with appropriate referrals to pain specialists including psychiatry.

Awareness is growing that chronic pain has a neurobiological basis and is a common complication of surgery. As the field specialized in procedures and surgeries involving the most densely innervated tissue in the body, there is great value in refining our understanding of both acute and chronic pain complications in ophthalmology. A more systematic and comprehensive approach to assessing pain is the first step toward innovative approaches to management and prevention of this important complication.

## Author Contributions

EM conceived of and directed the manuscript. MM-B drafted the initial manuscript. MM-B and EM wrote the final manuscript. All authors contributed to the article and approved the submitted version.

## Funding

The David Borsook Project, supported by the Cathedral Fund (awarded to EM).

## Conflict of Interest

The authors declare that the research was conducted in the absence of any commercial or financial relationships that could be construed as a potential conflict of interest.

## Publisher’s Note

All claims expressed in this article are solely those of the authors and do not necessarily represent those of their affiliated organizations, or those of the publisher, the editors and the reviewers. Any product that may be evaluated in this article, or claim that may be made by its manufacturer, is not guaranteed or endorsed by the publisher.
